# Site‐specific risk assessment enables trade‐off analysis of non‐native tree species in European forests

**DOI:** 10.1002/ece3.8407

**Published:** 2021-12-15

**Authors:** Anja Bindewald, Giuseppe Brundu, Silvio Schueler, Uwe Starfinger, Jürgen Bauhus, Katharina Lapin

**Affiliations:** ^1^ Department of Forest Conservation Forest Research Institute of Baden‐Württemberg (FVA) Freiburg Germany; ^2^ Chair of Silviculture University of Freiburg Freiburg Germany; ^3^ Department of Agricultural Sciences University of Sassari Sassari Italy; ^4^ Austrian Research Centre for Forests BFW Vienna Austria; ^5^ Julius Kühn‐Institut (JKI) Federal Research Centre for Cultivated Plants Braunschweig Germany

**Keywords:** climate change, context dependence, evidence base, forest management, Invasive alien trees

## Abstract

Non‐native tree species (NNT) are used in European forestry for many purposes including their growth performance, valuable timber, and resistance to drought and pest or pathogen damage. Yet, cultivating NNT may pose risks to biodiversity, ecosystem functioning, and the provisioning of ecosystem services, and several NNT have been classified as invasive in Europe. Typically, such classifications are based on risk assessments, which do not adequately consider site‐specific variations in impacts of the NNT or the extent of affected areas. Here, we present a new methodological framework that facilitates both mitigating risks associated with NNT and taking advantage of their ecosystem services. The framework is based on a stratified assessment of risks posed by NNT which distinguishes between different sites and considers effectiveness of available management strategies to control negative effects. The method can be applied to NNT that already occur in a given area or those NNT that may establish in future. The framework consists of eight steps and is partly based on existing knowledge. If adequate site‐specific knowledge on NNT does not yet exist, new evidence on the risks should be obtained, for example, by collecting and analyzing monitoring data or modeling the potential distribution of NNT. However, limitations remain in the application of this method, and we propose several policy and management recommendations which are required to improve the responsible use of NNT.

## Graphical abstract

A new methodological framework for site‐specific risk assessment facilitates mitigating possible negative impacts of non‐native tree species while using their ecosystem services in European forests. The method is based on a stratified assessment of risks posed by non‐native tree species that distinguishes between different ecosystem types and considers the effectiveness of available management strategies to control negative impacts. To improve the practical value of non‐native tree species risk assessments, we propose six recommendations that address forest and environmental policy as well as forest management.

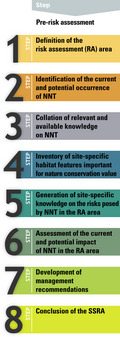

## 1 INTRODUCTION

For centuries, non‐native tree species (“NNT”) (Box 1) have been introduced to and cultivated in Europe, and now 4% of the European forest area is covered by more than 150 NNT (Brus et al., 2019). The major drivers for the use of NNT in forests are the economic benefits linked to their often better growth performance, timber properties, and pest resistance in comparison to native tree species (Pötzelsberger, Spiecker, et al., 2020). NNT are valued for their contribution to diversifying the portfolio of commercial native species (Sjöman et al., 2016; Willoughby et al., 2007), and particularly with regard to climate change, their use is recommended to increase forest resilience to drought as well as pest and pathogen damage (Bauhus et al., 2013; Bolte et al., 2009; Đodan et al., 2018; Thurm et al., 2018).

The downside of the use of NNT are possible negative effects on biodiversity and ecosystem services, such as the reduction of the protective function or productivity of forests, and negative effects on human well‐being. Of particular concern from a conservation perspective are NNT that spread from cultivated sites into protected areas, where they can potentially have “negative impacts” (Box 1) (Campagnaro et al., 2018). Such NNT are considered “invasive species” (Box 1) in Europe (Rejmánek & Richardson, 2013; Richardson & Rejmánek, 2011). Invasive species in general are known to cause high costs in terms of direct environmental and socioeconomic damage, as well as in terms of management efforts required to counteract such negative impacts or restore ecosystems (Angulo et al., 2021; Haubrock et al., 2021). Together with invasive non‐native species from other taxonomic groups, NNT are consequently regulated in the environmental, forestry, or plant health sectors. When NNT are evaluated as invasive, they are included in national lists of harmful/restricted species (e.g., Nehring et al., 2013; Pergl et al., 2016) or in regional, national, or European Union legislation (Pötzelsberger, Lapin, et al., 2020). These legal instruments generally follow a “blacklisting” approach, that is, all species causing negative effects are explicitly listed and either restricted or completely banned. For example, the use of *Ailanthus altissima* (Mill.) Swingle) is prohibited within the EU where it has been declared an invasive species of Union Concern according to Regulation (EU) No. 1143/2014 (Commission Implementing Regulation (EU) 2019/1262 of 25 July 2019). The negative impacts of NNT, however, are related not exclusively to tree‐specific characteristics but also to the specific context, that is, eco‐climatic site characteristics, co‐occurring vegetation, local fauna, propagule pressure, and cultivation or management techniques, all of which influence ecosystem sensitivity to NNT (Bartz & Kowarik, 2019; Sitzia et al., 2015; Wardle & Peltzer, 2017). Impacts are thus predominantly a product of species traits and site features (Sapsford et al., 2020). For example, black locust (*Robinia pseudoacacia* L.) can have significant negative impacts in open grassland while posing much lower or no risk in closed forest (Meyer‐Münzer et al., 2015). The risks of NNT may also change with different life stages. For example, the establishment potential of red oak (*Quercus rubra* L.) decreases in a beech forest during forest succession because it is outcompeted by other species (Nagel, 2015). As a consequence, context‐dependent abiotic and biotic constraints (Sapsford et al., 2020), as well as species‐specific potentials to establish or persist should always be considered in decisions about the use of NNT (Vor et al., 2015).

Some NNT are listed as invasive species in many European countries given their perceived or actual negative impacts based on the results of a “risk assessment” (Box 1). However, the methods applied in risk assessments across Europe were developed for different purposes and thus differ significantly in their approaches and outcomes (González‐Moreno et al., 2019; Matthews et al., 2017; Roy et al., 2018). Deficiencies in applying risk assessment methods to NNT have recently been highlighted as well. First, risk classifications are inconsistent due to the lack of a pan‐European protocol and are, therefore, not a reliable decision‐making support regarding NNT across country borders (Bindewald et al., 2020). Second, local observations of negative impacts are often extrapolated to larger spatial scales by providing a single absolute risk category—typically “potentially invasive” or “invasive” (Bartz & Kowarik, 2019). Risk assessments, therefore, do not sufficiently account for temporal and site‐specific variations of impacts, and they rarely consider the extent of the area impacted (Bindewald et al., 2020). In addition, little or no information is generally provided about the “sites” (Box 1) included in the risk assessment, and the methods thus fail to increase our knowledge about the context‐dependent drivers of NNT impacts (Sapsford et al., 2020). Third, the precautionary principle is typically applied leading to a classification of invasiveness based on the worst‐case scenario without taking into account all the available ecological studies (Strubbe et al., 2019). Fourth, assessment results provide little guidance on how to mitigate negative impacts, whereas an identification of sensitive ecosystems would be required to design cost‐efficient control strategies (Verheyen et al., 2007). Consequently, commonly used methods may have little practical relevance for forest and “risk management” (Box 1) (Bayliss et al., 2013; Wilson et al., 2014); on the contrary, they can exacerbate conflicts of interest regarding the use of NNT (Ammer et al., 2016; Dickie et al., 2014).

Since NNT can have benefits and disadvantages, a twofold conflict arises: Without considering sensitivity of ecosystem to NNT, using potentially invasive trees may lead to severe damage. Yet, a blacklisting approach alone would exclude potentially beneficial NNT without clear evidence that this damage occurs within the regions of interest. It is, therefore, important to provide a tool supporting decision systems with regard to the selection of sites, NNT, and silvicultural methods to control risks while taking advantage of the ecosystem services certain NNT provide (Dehnen‐Schmutz, 2011; Sjöman et al., 2016). Here, we developed a new methodological framework for site‐specific risk assessment (“SSRA”) (Box 1) that takes concrete ecosystem characteristics into account. We propose step‐by‐step guidance to provide research institutes, forest enterprises, conservation managers, and local and national authorities with a framework for integrating risk mitigation into forest management. While our focus lay on NNT already used or planned for use in European forestry, the idea of such a more practice‐oriented risk assessment for trees represents a potential solution for other taxa and regions worldwide as well. Specifically, we aimed to develop a framework enabling:

- assessment of the spatial and temporal risks of NNT, including the identification of sensitive ecosystems potentially at risk from NNT;
- strengthening of the evidence base by collecting and analyzing quantitative data in a structured, replicable, and transparent manner;
- suggestion of management approaches to mitigate (potential) negative impacts at specific sites while using promising NNT in other locations;
- identification of site‐specific data regarding NNT occurrence, regeneration dynamics, competitiveness, and potential impacts that are needed to facilitate SSRA.

BOX 1 **Glossary of key terms**

- **NNT:** “Non‐native,” “alien,” “introduced,” “exotic,” “nonindigenous,” or “allochthonous” tree species whose presence is the result of human activity (Krumm & Vítková, 2016).
- **Invasive species:** Non‐native species that pose a threat to biological diversity (COP VI/23 CBD 2002), and/or to human well‐being (Diagne et al., 2021).
- **Negative impacts of NNT:** Undesired ecological or socioeconomic effects associated with NNT. In Europe, four environmental impact mechanisms have been related to NNT (Pötzelsberger, Spiecker, et al., 2020): competition, hybridization, disease transmission, and alteration of the structure and function of ecosystems (Blackburn et al., 2014).
- **Risk:** The likelihood of negative impacts associated with NNT introduction, establishment, and/or spread and the magnitude of their consequences (ISPM 2, FAO, 2019a). Includes uncertainty regarding the actual effects, even for NNT for which data are considered adequate.
- **Risk assessment:** A standard method for evaluating negative impacts associated with the introduction, establishment, and spread of a non‐native species. The assessment serves as the information basis for prioritization of risk management and risk communication (ISPM 11, FAO, 2019b).
- **Risk management:** A method for analysis, identification, implementation, and communication of appropriate management options to reduce the risk posed by invasive species (ISPM 11, FAO, 2019b).
- **Site**: A location, habitat, or ecosystem type characterized by a specific assemblage of species, a specific abiotic environment (Bland et al., 2018), and a specific objective of management (Nyssen et al., 2016). If there are multiple different sites (e.g., forest communities), it may be useful to aggregate them into certain groups (e.g., major forest types) that could be relevant for understanding the context‐dependence of impacts (Bindewald et al., 2021).
- **Site‐specific risk assessment (SSRA)**: A stratified assessment of risks posed by NNT, which distinguishes between different locations, habitats, or ecosystem types (herewith defined for the first time).

## 2 MATERIALS AND METHODS

### 2.1 Identification of steps

First, we identified the basic components for the application of SSRA, that is, the aim, technical description, underlying principles, and expected outcomes as well as the method to be applied and the recommended data type to be used. Second, each step was formulated individually, and the other steps follow a logical order, while the major potential limitations for each step were identified (Table 1). Third, a number of theoretical scenarios were discussed among the authors to identify risk management decisions to be made.

### 2.2 Expert and stakeholder validation

Within a period of 3 months from September to November 2020, four online workshops for the validation of the SSRA for European forest ecosystems were conducted. One workshop with the project partners and observers of the INTERREG Alpine Space project ALPTREES, and three workshops with interdisciplinary groups of experts, public authorities, and stakeholders from the areas of forest conservation, silviculture, landscape planning, and nature conservation from Austria, Slovenia, France, Italy, and Germany. The workshop participants were encouraged to evaluate each step of the SSRA and suggest improvements. The discussions were structured into open consultation questions on the regional needs for SSRA as well as its applicability, the identification of the respective user groups, and the implementation strategies for policies and forest management actions following the SSRA. The expert and stakeholder responses were incorporated into the development of the SSRA framework.

## 3 RESULTS

**FIGURE 1:**
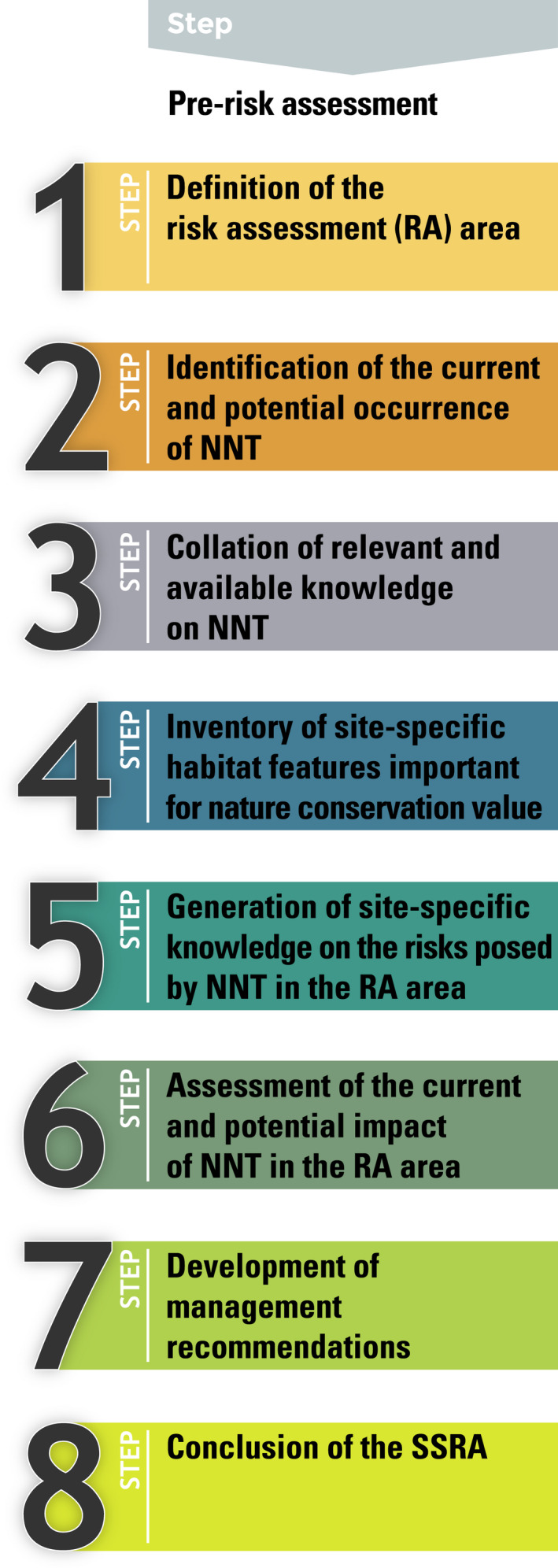
Overview of the steps of the site‐specific risk assessment to assess risks and management options associated with non‐native tree species; NNT, non‐native tree species; RA, risk assessment; SSRA, site‐specific risk assessment

As a result of the workshops as well as expert assessments, we propose a new SSRA, which comprises eight steps (Figure 1) that follow a pre‐assessment stating the reasons for its application. For each step, certain target information and data need to be collated (Tables A1, A2, A3 in the Appendix 1). Steps 1–4 are based on existing knowledge, while new knowledge is generated in Steps 5–7. In Step 8, key findings of Steps 1–7 are summarized. Steps 1–6 should be regarded as the first important steps enabling identification of sensitive ecosystem types or other spatially explicit areas (potentially) threatened by NNT. In Table 1, we summarize the key information and limitations linked to the data type required to complete each step of the SSRA.

**TABLE 1:** Key information linked to the respective data types and required to complete each step of the site‐specific risk assessment (SSRA)

SSRA Step	Key information	Recommended data source or data types	Method	Limitations
1	Area under assessment	National or regional boundaries, smaller political administrative units.	Expert‐based and remote sensing tools.	Spatial precision of the mapping tools available.
2	Occurrence of NNT	Verified identifications from vegetation distribution maps, national and regional forest inventories, forest reports, tree censuses, research or citizen science projects.	Literature and data review, screening of species observation databases.	Availability of monitoring data.
3	NNT‐specific and site‐specific information	Literature sources with information on the ecology, impact, management, extent and distribution of NNT (Table A1), for example, reports of forest inventories or research projects, information compilation from climatically similar regions, (inter)national legally binding prohibited invasive species lists, (inter)national, or regional lists of invasive species; already implemented risk or impact assessments of NNT.	Comprehensive literature and data review.	Poor evidence base of negative impacts or general lack of information; impact reports of low confidence level (IUCN, 2020); incoherent uses of the term “invasive”; inconsistent approaches to assessing invasiveness.
4	Inventory of site‐specific habitat features	Literature sources with information on valuable habitat features potentially threatened by NNT (Table A2), for example, IUCN Red List of Threatened Species, habitat types and species protected under regional, national, and European conservation acts; (regional) habitat mappings, assessment reports of habitat types, remote sensing data.	Comprehensive literature and data review.	Lack of data on features to assess the nature conservation value of the area; lack of site‐specific information indicating potentially affected habitat features.
5.	Site‐specific knowledge on NNT	Existing monitoring data, for example, national or regional forest inventories, or newly collected data on NNT recruitment and impacts on biodiversity and provisioning of ecosystem services (Table A3).	Data collection and analysis, field data collection protocols including local expert estimations to assess socioeconomic parameters, species distribution modeling.	Lack of resources to conduct a field survey, lack of trained staff, lack of available monitoring data; limited model performance due to nonanalogous climate in the RA area or unknown biotic interactions.
6	Current and potential impact of NNT	All information gathered in Steps 3–5.	Classification and evaluation of the evidence base of collected information (Strubbe et al., 2019); impact assessment for different sites, for example, (S)EICAT (Blackburn et al., 2014), expert‐based identification of high‐ or low‐risk NNT in a regional perspective (Figure 2).	Lack of references and evidence for the actual impacts and long‐term effects of NNT, particularly in sensitive ecosystems.
7	Management recommendation	Measurement recommendations based on research findings of Steps 3–6: species distribution and characteristics, sensitive ecosystems, dispersal distances, available resources described in management plans or reports of local administrations, NGOs, or forest enterprises (Tables A2 and A3).	Synopsis.	Weighing the risks of NNT to biodiversity or human well‐being on the one hand and the provisioning of ecosystems services on the other; changes in management may be controversial and require careful communication.
8	Conclusion of the SSRA	Summary of the key findings, including the objective of the SSRA, potential negative impacts for the different sites, limitation of the results, management recommendations as well as the level of uncertainty in the assessment.	Report.	Unclear communication of results may lead to generalization of impacts and thus to problematic errors in invasive species listing and in the communication of risks among stakeholders.

Abbreviations: NNT, non‐native tree species; RA, risk assessment.

### 3.1 Principles

Our framework is guided by widely accepted risk assessment standards (Roy et al., 2018) and follows the major underlying principles:

1. **Transparency and tracking of uncertainty:** All underlying data used to assess risks associated with NNT must be prepared and discussed in terms of their quality, robustness, and relevance to the area being studied to provide a solid evidence base for further communication.
2. **Evidence‐based decision support:** The evaluation should be based as much as possible on the analysis of quantitative data and ensure reliability and repeatability.
3. **No extrapolation of site‐specific results:** The results of the SSRA are applicable to the respective site only (as defined here) and cannot be transferred or generalized to any other site without evaluation of the corresponding site‐specific information.
4. **Restricted time frame:** The results of the SSRA are only valid for a certain period of time because ecosystems and the resident species communities are dynamic, for example, due to climate change, disturbances, or land‐use changes (Kulakowski et al., 2017). In addition, dynamics of the NNT may vary during the invasion process, as some populations may successfully establish or spread while others may fail to become invasive, depending on prevailing site conditions and NNT‐specific plasticity or fitness (Blackburn et al., 2011).

#### 3.1.1 Pre‐assessment

Any risk assessment can be costly and time‐consuming (Helland, 2009). To be conducted efficiently, a pre‐assessment, therefore, aims to identify the needs, motivations, goals, and expected benefits of the SSRA. This preliminary step ensures the consistency as well as the transparency (Liem, 2008; Schreider, 2008) of the risk assessment. Therefore, prior to a SSRA, it is necessary to state explicitly why the assessment is necessary and beneficial for a specific area. It should be clarified whether the target is to assess the risks of one or several specific preselected NNT and whether the NNT are present or not yet present in the risk assessment area.

#### 3.1.2 (STEP 1) Defining the risk assessment area

The SSRA can be performed at different spatial scales, that is, at the local, regional, and landscape level. The selected area may be, for example, a biosphere reserve, a specific region, or even a country, depending on the objective of the SSRA and the time and resources available to conduct the assessment. The assessor should identify the risk assessment area (FAO, 2019b) and possibly display it on a map.

#### 3.1.3 (STEP 2) Identifying the occurrence of NNT in the risk assessment area

The aim of Step 2 is to assess the presence of all NNT or the presence of a preselected NNT in the risk assessment area. The presence of NNT can be asserted by monitoring data, observations, and personal communication with local experts or stakeholders as well as other sources. NNT not yet reported to occur in the area can also be identified in Step 2 depending on the overall objective of the SSRA.

#### 3.1.4 (STEP 3) Collating the available relevant knowledge on NNT

Step 3 is conceived as a desk survey aiming at the collation of relevant existing knowledge on the selected NNT and, if it is present in the risk assessment area, its extent and distribution pattern. Available knowledge on the NNT should be collated into the following information categories: ecology, extent and distribution, impact, and management of the NNT (Table A1). In addition, all relevant information on the legal status of NNT in the risk assessment area and any applicable legal restrictions should be gathered as well (Brundu et al., 2020). This includes legally binding international, national, or regional regulations and/or legislation concerning NNT, for example, with the aim of preventing the use of (potentially) invasive NNT (see Pötzelsberger, Lapin, et al., 2020 for Europe). For the desk survey, we recommend distinguishing between information that is specific to the NNT (NNT‐specific) and can, therefore, be collected from sources not necessarily related to the risk assessment area, on the one hand, and information specific to the assessed area (site‐specific), on the other (Table A1).

#### 3.1.5 (STEP 4) Inventory of the site‐specific habitat features of high conservation value

Step 4 is conceived as a desk survey aiming to define the area under threat of NNT invasion within the risk assessment area (ISPM no. 5 FAO, 2019b). Areas with specific unique features of high conservation value in which NNT are already present or which are located within dispersal distance of NNT stands are particularly relevant. However, even sites that do not appear to be at risk may be relevant, as uncertainties owing to a lack of studies and monitoring data may remain (Latombe et al., 2019). We recommend to gather this information with special consideration for endangered habitats (Janssen et al., 2016), the status of biodiversity and ecosystem services, threats, and management objectives (Table A2).

#### 3.1.6 (STEP 5) Generating site‐specific knowledge on NNT

If adequate site‐specific knowledge on NNT does not yet exist for the risk assessment area, the aim of step 5 is to obtain new evidence on the risks of NNT, with a particular focus on habitat features for nature conservation value. If several NNT have been selected for the SSRA, Steps 5–7 should be completed for each individual tree species. Based on a list of parameters already identified and collected in various ecological studies (Table A3), we propose three promising methods to generate new knowledge:

##### Inventories

This approach is based on already existing systematically collected monitoring data and focuses on NNT that are sufficiently abundant to be captured in regular inventories. The aim is to quantify spatial and temporal patterns of NNT occurrence across different ecosystems and protected areas to prioritize control measures (e.g., Rouget et al., 2002; Shackleton et al., 2017). Where available, data on NNT natural regeneration should be assessed across different sites to identify those ecosystems that facilitate the establishment of NNT. In addition, repeated inventories can be used to determine how the state of NNT is developing over time. Such data can be derived from regional and national forest inventories (NFI), regional datasets covering protected areas (e.g., Bindewald et al., 2021; Oswalt et al., 2015; Steinmetz & Bauhus, 2016; Verheyen et al., 2007; Wagner et al., 2017), or from assessment reports of habitats valuable for nature conservation (e.g., EU Habitats Directive, Campagnaro et al., 2018). However, the usefulness of this method is limited to NNT that have already reached a certain distribution (Klemmt & Neubert, 2011). Besides, if the assessed area is too small, the number of observations collected may not be sufficient to allow assessments with acceptable precision (Breidenbach & Astrup, 2012).

##### Field surveys

If resources are available, new data on existing populations of the NNT can be collected in dedicated field surveys. This method can be applied to any NNT occurring within the risk assessment area irrespective of the extent of its distribution. Based on the identified knowledge gaps (Steps 3 and 4), such surveys may use different methodological approaches covering varying spatial scales. Relevant sites for further data acquisition in a field survey must be justified, for example, by selection of sensitive ecosystems. Proposed methods to quantify the risks posed by non‐native plants in the field are manifold. A possible motivation for such a study could be to obtain knowledge on the composition of a regenerating community at smaller spatial scales. In this case, information about the current stage of NNT establishment, dispersal distances, and site‐specific factors that influence NNT recruitment can be collected in plots or transects (Dyderski & Jagodziński, 2018; Nygaard & Øyen, 2017; Woziwoda et al., 2018). To assess local effects on biodiversity, additional ecological data such as cover and diversity of the herb layer may be collected as well (Woziwoda et al., 2014). To survey spatial and temporal changes in ecological impacts, experimental plots can be installed in different sites (Barney et al., 2015). As some NNT can alter ecosystem processes (e.g., nitrogen fixation causing eutrophication), studies may focus on assessing effects on nutrient cycling (Hellmann et al., 2017; Rascher et al., 2012). Impacts of the admixture of NNT on forest biodiversity can be studied by comparing stands with varying proportions of NNT, as has been proposed for Douglas fir (*Pseudotsuga menziesii* (Mirb.) Franco) (Wohlgemuth et al., 2019).

##### Modeling

This toolbox can be used whenever the goal of the SSRA is to guide land and forest managers regarding long‐term planning. Various types of ecological models are available to simulate the potential distribution (Boiffin et al., 2017; Chakraborty et al., 2019), growth (Landsberg et al., 2003), regeneration (Eberhard & Hasenauer, 2018), spread (Nathan et al., 2011), or population dynamics (Sebert‐Cuvillier et al., 2007) of NNT in their new environment. Among these, species distribution models are the most widely used (Boiffin et al., 2017; Chakraborty et al., 2019; Richardson et al., 2010) because they relate the species' potential distribution to climate and land‐use change (Petitpierre et al., 2012), two important drivers of NNT spread (Camenen et al., 2016; Lenda et al., 2018; Nadal‐Sala et al., 2019). They can be applied to NNT that are already present (e.g., Oswalt et al., 2015; Verheyen et al., 2007) or those not yet present in the risk assessment area (Puchałka et al., 2021). If NNT are not yet present, distribution models can help to support pre‐entry risk‐screening tools to inform managers of potentially invasive NNT (Weber & Gut, 2004). However, the application of species distribution models that have been calibrated with information from its native range outside of that range requires careful validation (Boiffin et al., 2017; Camenen et al., 2016).

#### 3.1.7 (STEP 6) Assessment of the current and potential impacts of NNT

The aim of Step 6 is to assess the site‐specific risks of NNT in the risk assessment area, that is, the likelihood and magnitude of negative impacts on the site and/or any protected assets (to be clearly and explicitly identified). If the knowledge regarding a certain NNT remains data deficient after Steps 3–5, its impacts cannot be evaluated. Still, monitoring may be recommended under Step 7. We recommend assessing the (potential) impacts in three substeps as described in the following:

##### Evidence ranking

All collated data and information on current and potential impacts should be classified and ranked by their level of evidence and relevance to the risk assessment area (Binkley & Menyailo, 2005; Kohler et al., 2020; Strubbe et al., 2019). With this in mind, evidence in peer‐reviewed studies should be considered more reliable than information gained from other sources (e.g., expert opinions, field excursion reports), and data collected in the risk assessment area should be considered more relevant than data from other regions (Strubbe et al., 2019).

##### Impact assessment

This evidence base can be used to assess the magnitude of (potential) negative environmental or socioeconomic impacts for the different sites and sensitive ecosystems identified in previous steps. For example, the “(Socio)‐Economic impact classification of alien taxa” (EICAT, SEICAT) methodology can be helpful for quantifying, comparing, and prioritizing different impact mechanisms of NNT for different sites (Bacher et al., 2018; Blackburn et al., 2014; Lapin et al., 2021). These approaches follow a categorical system ranging from minimal to massive impacts and take the reversibility of impacts into account. Any evidence of NNT natural regeneration outside of sites demarcated for cultivation should be examined to identify sensitive ecosystems that might be affected (Brundu et al., 2020). In the absence of better information on actual impacts, any potential impact should be carefully considered, considering possible successional dynamics of the NNT on site as well as tree‐specific characteristics such as spread potentials, shade tolerance, and competitiveness (Table A1). Both the extent of the impact, that is, the number of species and the size of the area affected, and the value of affected goods should be considered, that is, species and habitats of importance for conservation at the local, regional, national, or European level (Table A2). Since NNT can replicate “invasive behavior” under environmentally similar conditions (Essl et al., 2010), experience from other areas may be considered, if obtained under comparable conditions or in similar ecosystems.

##### Decision tree

**FIGURE 2:**
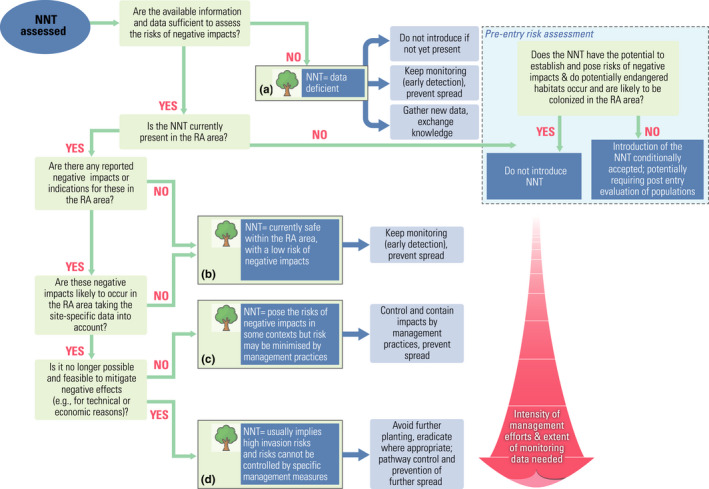
Decision tree demonstrating practical application of the site‐specific risk assessment; NNT, non‐native tree species; RA, risk assessment; SSRA, site‐specific risk assessment

The information obtained in the previous stages can be used to apply the SSRA decision tree (Figure 2). NNT are classified into one of four groups with different management options in the risk assessment area at the time of the SSRA: (a) NNT data deficient, (b) safe NNT expected to pose no risks, (c) NNT that can pose risks in some environmental contexts, but risks can be kept low, and (d) NNT expected to pose high risks that cannot be controlled. The reversibility of any negative impact and the options for controlling populations through available management measures (Table A1) (Vor et al., 2015) are important criteria for assignment to the four groups:

- **NNT data deficient**. NNT for which information about their life‐history strategies, phylogenetic or taxonomic status, adaptability with concern to eco‐climatic factors, or other characters affecting their ability for unintended dispersal is scarce or entirely lacking, pose unknown risks. If such species and/or cultigens are already present in the risk assessment area, no urgent measures need to be taken, but their stands should be carefully monitored. Species and/or cultigens with considerable data deficiency should not be imported and widely planted, if they are not yet present in the risk assessment area. If certain NNT seem very promising for future use in forests, more information about such species should be gathered under low‐risk conditions (e.g., in their native range or by establishing sound trials or sentinel gardens) (Carrillo‐Gavilan & Vila, 2010; Fanal et al., 2021). For species that are well‐studied with respect to the abovementioned characteristics but not yet present, the decision whether or not to import and plant them should be based on a classic pre‐entry risk assessment procedure (e.g., Branquart et al., 2016; Křivánek & Pyšek, 2006; Verbrugge et al., 2019).
- **Currently safe NNT**. For NNT that are present and well‐studied, management decisions should focus on possible negative impacts in the risk assessment area. If no negative impacts could be identified, use of these NNT can currently be considered safe. However, to minimize potential risks, we recommend following accepted standards and guidelines when cultivating these tree species (e.g., Brundu & Richardson, 2016). NNT may still be used even if they have been considered invasive or have the potential to spread or have other negative effects, provided sites where negative impacts could potentially occur do not exist in the risk assessment area. Management decisions should be based on the question whether such impacts are likely to occur within the forest stands or in adjoining sensitive areas that may be reachable by the NNT. If such NNT are planted, their stands should be carefully monitored for any change in their behavior, such as unintentional spreading. Furthermore, NNT should be planted in mixtures with native trees to avoid any possible risks to forest biodiversity (Kriegel et al., 2021; Oxbrough et al., 2016; Wohlgemuth et al., 2021).
- **NNT whose risks can be controlled**. NNT with potentially negative effects on sensitive ecosystems present in the risk assessment area may still be relatively safe to use, if management practices exist that exclude or strongly control such risks—for example, by way of physical removal and silvicultural approaches, such as admixing competitive native trees, or establishing buffer zones around sensitive areas (Sitzia et al., 2015; Vor et al., 2015). Such NNT can be allowed for further use provided these measures are applied.
- **High‐risk NNT**. If the impacts caused by the NNT in the risk assessment area are not reversible or cannot be excluded by cost‐efficient management measures that are acceptable to both stakeholders and the public, the outcome is the recommendation to discontinue the use of this NNT. In addition, further risks should be mitigated, for example, by ensuring the best possible protection of sensitive ecosystems. An obvious option is to eradicate existing stands in protected areas and restore them after removal, but this may not always be feasible (e.g., black cherry (*Prunus serotina* Ehrh.), Nyssen et al. (2016)) or may cause undesirable side effects (Sitzia et al., 2015), so careful planning needs to be applied (Booy et al., 2017).

#### 3.1.8 (STEP 7) Management recommendations

The aim of Step 7 is to develop an action plan for the management of the NNT in the risk assessment area, which in addition to legislation‐driven decisions may include recommended measures for voluntary priority actions, local eradications, prevention of spread and establishment, and further monitoring for the entire assessment area or the sensitive ecosystems it contains. Based on these preceding steps species‐specific management objectives can be formulated (Alberternst & Nawrath, 2018). Depending on NNT characteristics and distribution pattern in the risk assessment area, management measures (e.g., eradication, population control) and related costs may not be achievable. For example, local eradications of populations of NNT with the ability to resprout or form root‐suckers such as black locust (*Robinia pseudoacacia* L.) may entail disproportionate costs (Meyer‐Münzer et al., 2015).

#### 3.1.9 (Step 8) Conclusion of the SSRA

The aim of Step 8 is to summarize the objective (as defined in the pre‐assessment) and the outcomes of the SSRA, especially those of Steps 5–7, for further communication including justification and limitation of the results, level of uncertainty (Clarke et al., 2020), and reasons for uncertainty in the assessment (Roy et al., 2018). The final deliverables of the SSRA should include a journal of the methodology applied, references of the data sources used, a NNT‐specific summary of the assessment decision, a spatially explicit action plan, and a timeframe for the recurrence of the SSRA. Furthermore, Step 8 can provide a cross‐evaluation of the assessed NNT. To guarantee the transparency and transferability of the SSRA to other areas, it is necessary to explicitly describe the identified sensitive ecosystems in the RA area, and the size of the area affected along with the types of data and methodological approaches employed to assess site‐specific risks, the name, affiliation, and expertise of the assessor.

## 4 DISCUSSION

Unlike previously developed risk assessment methods for non‐native plants (Bartz & Kowarik, 2019), the SSRA proposed here allows consideration of varying levels of ecosystem sensitivity to NNT and existing management options. Resembling the ideas of Sjöman et al. (2016) for different uses of NNT in cities, the SSRA decision tree allows for resolving conflicts of interest between nature conservation objectives and forest management goals, including climate adaptation and mitigation strategies. Overall, the framework for the SSRA aims to build a bridge between a precautionary approach toward introducing new risks and the possibility of continuing the use of NNT—including those with a potential of causing negative impacts. Refraining from using species that are neither present nor sufficiently well‐known is a typical element of a “whitelisting” approach (“guilty until proven innocent”), which seems reasonable considering that specific information is often lacking even for commonly planted NNT (Magona et al., 2018). Otherwise, the spatial and temporal variation of impacts and the potentially existing options for management allow the use of NNT under certain circumstances even in the case of blacklisted species (Starfinger & Kowarik, 2016). Overall, the framework of the SSRA still suffers from a number of limitations in its application, communication, management, and policy implementations.

### 4.1 Implementation

The wide range of possible sizes of risk assessment areas has a large influence on the accuracy and efficiency of the method. Given that only limited time and resources are usually available, results are expected to be coarse in resolution when the SSRA is applied at the scale of a large country or region. In this case site‐specific management recommendations will be difficult to make. Conversely, if the assessed area is very small (e.g., a protected habitat), analysis of invasion patterns across a range of different sites is hardly possible, and the SSRA thus cannot distinguish between ecosystems with varying sensitivity to invasion. In principle, the result of the SSRA is valid only for a certain period of time, as the environmental context for the risk assessment can change (sometimes rapidly). In fact, the structure and composition of forest ecosystems, and thus the dynamics of NNT populations can be influenced by natural disturbances (Seidl et al., 2014), such as severe drought events (e.g., Frischbier et al., 2019; Messinger et al., 2015), storm damage (e.g., Albrecht et al., 2013), wildfires (Silva et al., 2011), or pests and diseases (Ennos et al., 2019), as well as by succession and anthropogenic disturbances, such as logging or clearing (Grindean et al., 2019). Establishment of more light‐demanding NNT such as Douglas fir, for example, may be promoted since natural disturbances are likely to increase in European forests under predicted climate change conditions (Seidl et al., 2017). Yet, populations of NNT will also disappear from parts of the landscape where they are deemed unsuitable, for example, because of extreme drought (Rigling et al., 2016). A further weakness is that the evidence base of SSRA may depend on the importance and extent of NNT. Data and research findings on NNT are typically available for species that have begun to cause noticeable impacts and are already widespread after having been introduced to an area a long time ago, such as tree of heaven in many regions in Europe (Sladonja et al., 2015). Especially in forest inventories, information on NNT is usually only found for species that have been cultivated for some time, such as Douglas fir, red oak, or Japanese larch (*Larix kaempferi* (Lamb.) Carrière) in Germany, although these species together still make up only 5% of the entire forest (BMEL, 2012). Therefore, large‐scale data are not helpful for early detection of NNT and essential knowledge for rapid response efforts is lacking (Jarnevich et al., 2006).

### 4.2 Communication

Stakeholder engagement on the risks and management options of NNT in different regions is strongly recommended (Brundu et al., 2020), particularly when the species is considered both useful for forestry and harmful to ecosystems (e.g., Vítková et al., 2017). When communicating the invasive potentials of NNT, there remains a risk of false interpretation of the SSRA results, for example, when the assessed area is very small and/or results are based only on a single field study. Clear communication, therefore, should be practiced when sharing the results with policymakers, practitioners, and the public. Communication of the SSRA results should always occur in the context of the risk assessment area. In addition, we propose avoiding use of the term “invasive” as a final category resulting from the SSRA, since no non‐native species is invasive in all areas it has been introduced to (Heger, 2016) and invasion terminology is often used inconsistently (Falk‐Petersen et al., 2006). Instead, we recommend communicating the magnitude and probability of risks posed by NNT at different sites and referring to the specific stage of a biological invasion, that is, the establishment, spread, or impact (Colautti & MacIsaac, 2004). For example, concern has been expressed that red oak competes with native tree species in semi‐natural oak forests in Germany (Nehring et al., 2013). Although a recent study showed that red oak has spread into certain protected oak forest communities in southwest Germany, there is still insufficient data on the stage of establishment and the actual (long‐term) impacts on biodiversity in these sensitive ecosystems to draw firm conclusions, for example, on its competitiveness (Bindewald et al., 2021).

### 4.3 Forest management

The implementation of forest management can noticeably help to reduce or prevent undesired effects of NNT (Sitzia et al., 2015). The choice of management measures depends on the impact the NNT has on local management goals. For example, if forest succession can be accepted as a strategic option, forest managers can apply silvicultural techniques to alter interspecific competition, thereby suppressing unwanted regeneration of certain NNT and promoting the desired tree species composition (Nyssen et al., 2016). While such common silvicultural techniques are already widely used in European forestry (e.g., Meloni et al., 2016; Nagel, 2015), the situation seems to be different for areas of conservation value. Since more than half of all Natura 2000 areas in Europe consist of forests, and because established NNT can pose high risks to biodiversity in such ecosystems (Campagnaro et al., 2018; Sitzia et al., 2012), forest managers carry great responsibility for preventing or mitigating those risks. Eradication may not be practical for various reasons. Measures can be very expensive—combating black cherry in the Netherlands has caused an estimated expenditure of € 200 million, for example (Nyssen et al., 2016). What is more, eradication efforts often deliver limited success when NNT are able to reproduce through coppice shoots or root suckers, such as red ash (*Fraxinus pennsylvanica* Marsh) (Zacharias & Breucker, 2008). The feasibility of eradication measures should, therefore, be weighed against the risks (Booy et al., 2020) and the endangerment of the subject of protection. It is essential that the management of forest stands with NNT respects sensitive ecosystem types in the landscape, for example, by establishing appropriate buffer zones (e.g., 300 m for Douglas fir in southwest Germany) (ForstBW, 2014).

### 4.4 Knowledge and research gaps

The application of the SSRA cannot replace major research activities and monitoring programs, which can provide necessary data for identifying and mitigating threats from NNT (Bastrup‐Birk & Schuck, 2016). Although there is an increasing availability of data on tree species' natural distributions and tree occurrences globally, local and regional data are often lacking. In addition, the spatial accuracy of global data may be limited (Mauri et al., 2017; Serra‐Diaz et al., 2018), creating a shortfall in monitoring and the regional application of global species distribution models.

Furthermore, there is a lack of long‐term empirical data on the ecological impact and evolution of (potentially) invasive NNT in Europe (Krumm & Vítková, 2016). Recent reviews on risks associated with the use of NNT in European forestry have identified several gaps that could direct future research (e.g., Felton et al., 2013; Kjaer et al., 2014; Pötzelsberger, Spiecker, et al., 2020; Schmid et al., 2014). For example, regional knowledge gaps exist with regard to changes in typical species composition associated with the establishment of NNT in rare and endangered forest communities (e.g., Bindewald & Michiels, 2018). Moreover, comparisons of communities of forest‐dwelling taxa in stands or individuals of non‐native tree species with stands or individuals of other tree species are not very helpful (Bauhus et al., 2017). Such differences in biodiversity attributed to NNT occurrence are largely predictable when the tree species differ substantially regarding their traits and habitat attributes or when NNT stands are compared with nonforest ecosystems like grasslands (e.g., Finch & Szumelda, 2007; Horák et al., 2021; Kühnel, 1995). Much more relevant questions for silvicultural management of NNT are: (1) what is the site‐specific establishment potential of NNT widely used throughout Europe, and which sites are most sensitive? (2) What distances are required to establish effective buffer zones around sensitive ecosystems for the individual NNT? (3) To what degree can NNT be added to stands of other tree species without negatively affecting the viability of populations of native species at different spatial scales (e.g., Bollmann & Tschopp, 2016; Kriegel et al., 2021)? The last point in particular is important in relation to the widely accepted silvicultural strategy that forests should be mixed to spread risks and thus adapt to uncertain future disturbances (Ammer, 2019; Bauhus, Forrester, et al., 2017).

### 4.5 Additional policy and management recommendations

We are convinced that in addition to the use of the SSRA, changes in forest and environmental policy and forest management are required to improve the practical value of NNT risk assessments. For this purpose, we propose the following six recommendations:

1. Forest authorities must ensure that measures to minimize the risks of NNT are in place and fully integrated into guidelines for best management practice (e.g., Brundu et al., 2020). In this context, containment of NNT populations to areas set aside for their cultivation, for example, by setting exclusion zones for planting around sensitive areas, should be compulsory in regional or national forest management plans.
2. Silvicultural adaptation strategies should focus primarily on NNT that currently pose no risks, or NNT that pose risks only in some environmental contexts, and these risks can be controlled (NNT of group b and c, Figure 2). At the same time, these NNT need to be continuously monitored, for example, as part of forest inventories (Bindewald et al., 2021).
3. Forest authorities should assess and report the extent and distribution of NNT, as well as their actual or potential effects. This information should be shared among authorities to support efficient management options.
4. Introduction and promotion of new NNT should follow a pre‐entry risk assessment, and initial trials should be conducted under the guidance of agreed standards minimizing risks (Ennos et al., 2019).
5. Terminology on NNT should be harmonized across Europe to improve communication between different stakeholders and policymakers.
6. It is necessary to support research on the biology of NNT, their actual and potential distribution as well as their long‐term effects (both positive and negative) such as evolutionary interactions with native forest species.

## CONFLICT OF INTEREST

The authors declare no competing interests.

## AUTHOR CONTRIBUTIONS

**Anja Bindewald:** Conceptualization (equal); funding acquisition (supporting); investigation (lead); methodology (equal); writing – original draft (lead); writing – review and editing (lead). **Giuseppe Brundu:** Investigation (supporting); methodology (supporting); writing – original draft (supporting); writing – review and editing (supporting). **Silvio Schüler:** Investigation (supporting); methodology (supporting); writing – original draft (supporting); writing – review and editing (supporting). **Uwe Starfinger:** Investigation (supporting); methodology (supporting); writing – original draft (supporting); writing – review and editing (supporting). **Jürgen Bauhus:** Conceptualization (supporting); supervision (supporting); writing – original draft (supporting); writing – review and editing (supporting). **Katharina Lapin:** Conceptualization (equal); funding acquisition (lead); investigation (supporting); methodology (equal); supervision (lead); writing – original draft (supporting); writing – review and editing (supporting).

## Data Availability

Examples and references for the methodological framework of the site‐specific risk assessment are provided in Appendix 1.

## APPENDIX 1

**TABLE A1:** Collating the relevant and available knowledge on non‐native tree species (NNT) in Step 3; RA=risk assessment

Category	information level	Target information	Reference
Ecology	NNT‐specific	competiveness	IUCN (2020), Vor et al. (2015)
	NNT‐specific	invasion history elsewhere	Roy et al. (2018)
	NNT‐specific	regeneration potential: persistence of seed bank	Pyšek et al. (2012)
	NNT‐specific	regeneration potential: reproductive means (vegetatively via coppice shoots, root suckers)	Pyšek et al. (2012), Vor et al. (2015)
	NNT‐specific	regeneration potential: seed and propagule production	Parker and Gilbert (2007), Pyšek et al. (2012)
	NNT‐specific	spread potential: seed dispersal distances	Parker and Gilbert (2007), Vor et al. (2015)
	NNT‐specific	spread potential: spreading mechanisms	Parker and Gilbert (2007), Sladonja et al. (2015)
	NNT‐specific	taxonomy	Roy and Scalera (2014)
	NNT‐specific	tree growth and natural regeneration: soils, climate, light	Bindewald et al. (2020), Sitzia et al. (2015), Vor et al. (2015)
Extent and distribution	NNT‐specific	distribution range (native and introduced)	IUCN (2020)
	site‐specific	actual and potential distribution in the RA area	Rouget et al. (2002), Roy and Scalera (2014)
	site‐specific	extent of the current cultivation area of the NNT	CBD (2014), Pyšek et al. (2012)
	site‐specific	history in RA area: increase of naturalized populations	Haysom and Murphy (2003)
	site‐specific	history in RA area: temporal and spatial development in the abundance	Bindewald et al. (2021)
	site‐specific	history in RA area: the year of the first report of escape from cultivation	Kowarik (1995), Křivánek and Pyšek (2006), Pyšek et al. (2009)
	site‐specific	identification of existing databases with monitoring data	Bindewald et al. (2021)
	site‐specific	likelihood of establishment across different sites in the RA area	Bindewald et al. (2021), Vor et al. (2015)
	site‐specific	occurrence of NNT across different forest and land cover types	Wagner et al. (2017)
	site‐specific	pathways: escape from managed sites, unaided across borders	CBD (2014)
	site‐specific	pathways: frequency of movement along the pathways	Schrader and Starfinger (2009)
	site‐specific	pathways: means of intentional und unintentional spread	CBD (2014), Pyšek et al. (2012)
Impact	NNT‐specific	environmental impact mechanism with respect to biodiversity or ecosystem patterns and processes	Blackburn et al. (2014)
	NNT‐specific	hybridization: genetic dilution of native con‐generics through hybridization	Felton et al. (2013)
	NNT‐specific	native species displacement: changes in habitat provision for native taxa	Blackburn et al. (2014), IUCN (2020), Vor et al. (2015)
	NNT‐specific	native species displacement: competition with native species	Blackburn et al. (2014), IUCN (2020), Vor et al. (2015)
	NNT‐specific	native species displacement: potential to establish permanent populations	Branquart et al. (2016), Vor et al. (2015)
	NNT‐specific	pests and pathogens: likelihood of increasing the risk of outbreaks	EC (2018), Felton et al. (2013), Gossner (2016), Pötzelsberger et al. (2021)
	NNT‐specific	Positive effects on biodiversity: e.g. habitat provisioning for forest dwelling species	Bouget et al. (2021), Kriegel et al. (2021)
	NNT‐specific	Positive effects on provisioning, regulating and cultural ecosystem services: e.g. timber production, increased productivity of forests and carbon uptake, mitigation of natural hazards and climate regulation, soil formation, erosion control and other protective functions of forests, ecological and cultural benefits (e.g., ornamental trees)	Castro‐Díez et al. (2019), Dodet and Collet (2012), Roy and Scalera (2014), Vaz et al. (2018), Vaz et al. (2017)
	NNT‐specific	economic costs of invasive species: e.g. losses of biodiversity, reduced ecosystem services, the costs of controlling invasive species and mitigating their impacts, ecosystem restoration	Haubrock et al. (2021), Bacher et al. (2018), Haubrock et al. (2021), Kettunen et al. (2008), Pyšek and Richardson (2010)
	NNT‐specific	negative effects on human health and wellbeing: e.g. NNT pollen causing allergies in humans, NNT reducing the benefit of human–nature interaction	Bergmann et al. (2020), Castro‐Díez et al. (2019), Diagne et al. (2021)
	site‐specific	alteration of ecosystem processes: changes in nutrient cycling, trophic interactions, and in the water budget	Le Maitre et al. (2011), Roy and Scalera (2014), Sladonja et al. (2015), Vor et al. (2015)
	site‐specific	negative effects on regulating and provisioning ecosystem services: e.g., increase in fire, erosion, or avalanche risk, decrease of agricultural or forestry productivity; likelihood of losses of ecosystem services	Annighöfer et al. (2015), Castro‐Díez et al. (2019), Dickie et al. (2014), IUCN (2020)
	site‐specific	negative effects on cultural ecosystem services, e.g. recreation, aesthetics	Castro‐Díez et al. (2019), Vaz et al. (2018)
	site‐specific	likelihood of NNT‐induced decline in conservation status or value	EC (2018), Felton et al. (2013)
	site‐specific	native species displacement: modification of sensitive ecosystems	Felton et al. (2013)
Management	NNT‐specific	controlling and containing strategies: prevention of intentional introductions	EC (2018)
	NNT‐specific	controlling and containing strategies: prevention of unwanted dispersal	EC (2018), Sitzia et al. (2015), Vor et al. (2015)
	NNT‐specific	controlling and containing strategies: rapid eradication for new introductions	EC (2018), Vor et al. (2015)
	NNT‐specific	controlling and containing strategies: removal of unwanted regeneration	EC (2018)
	NNT‐specific	controlling and containing strategies: seed bank control	
	NNT‐specific	monitoring: Surveillance measures to support early detection	EC (2018), Wilson et al. (2014)
	NNT‐specific	silvicultural measures to reduce spread: Tree species selection, coppicing, maintaining or facilitating closed canopy, girdling	Sitzia et al. (2015)
	site‐specific	feasibility: acceptability to stakeholders, cost information, practicality, effectiveness, likelihood of re‐invasion	Booy et al. (2017), EC (2018)
	site‐specific	legal status incl. restrictions for management and use	Brundu et al. (2020), Pötzelsberger, Lapin, et al., 2020
	site‐specific	monitoring: regular and systematic monitoring, particularly in natural habitats	Monty et al. (2016), Wilson et al. (2014)
	site‐specific	management objectives and recommendations	Nyssen et al. (2016), Tree App

**TABLE A2:** Inventory of the site‐specific habitat features and nature conservation value of the risk assessment area in Step 4; NNT=non‐native tree species, RA=risk assessment

Category	Target information	Reference
Conservation management	Area of forest protected with the aim of conserving biodiversity	Forest Europe (2020)
	biodiversity indicator species for biodiversity	Oettel and Lapin (2021)
	Conservation and utilization of forest tree genetic resources	Forest Europe (2020)
	conservation management goals	IUCN (2017)
	identification of past and ongoing management actions	Forest Europe (2020)
	identification of monitoring data	Bindewald and Michiels (2018), Campagnaro et al. (2018)
	legal nature conservation status and restrictions	Habitats Directive (1992), Pötzelsberger, Lapin, et al., 2020
	management requirements	IUCN (2017)
	regional strategies and guidelines	Pötzelsberger, Lapin, et al. (2020)
	status (threatened or protected) of species or habitat under threat	IUCN (2017)
	umbrella species/ flagship species	Lõhmus et al. (2017), Naumov et al. (2018), Peura et al. (2018), Walentowski et al. (2013)
Forest management	area managed for seed production	Forest Europe (2020)
	current land use management description	Forest Europe (2020)
	ecosystem services important for forest management	Forest Europe (2020)
	intensity of forest management	Sitzia et al. (2015)
	Intensity of human influence	Sitzia et al. (2015)
Habitat description	abiotic constraints: elevation, soil types	Forest Europe (2020)
	abundance, species richness and evenness of native and non‐native tree species	Forest Europe (2020)
	area of regeneration within even‐aged stands and uneven‐aged stands	Forest Europe (2020)
	deadwood volume and diversity by tree species	Oettel and Lapin (2021)
	ecosystem services provided by forests	IPBES (2019)
	ecosystem services provided by tree species	Castro‐Díez et al. (2019), Hummel et al. (2017)
	geological features of significance (e.g., rocks, Karst, caves)	Kerner and Geisel (2017)
	habitat connectivity	Forest Europe (2020)
	habitat provisioning by tree species	Bütler et al. (2013), Kozak et al. (2018)
	plant species diversity	Avalos et al. (2006)
	presence of endemic species	Forest Europe (2015), Forest Europe (2020), Rivers (2019)
	structural diversity	Chmura (2020), Dyderski and Jagodziński (2020)
	tree species composition	Forest Europe (2015)
	tree species diversity	Schmitt et al. (2019)
Threats	Constraints	IUCN (2017)
	direct and indirect disturbances	Forest Europe (2015)
	drivers of Threats	IUCN (2017)
	effects of climate change	IPBES (2019)
	identify threatened ecological communities/invasible ecosystems	Catford et al. (2012)
	likelihood of NNT‐induced decline in conservation status	Campagnaro et al. (2018)
	likelihood of NNT‐induced decline in species and habitat under threat	Campagnaro et al. (2018)
	regeneration inhibiting factors	Forest Europe (2015), Forest Europe (2020)
	tree growth‐inhibiting influences	IUCN (2017)

**TABLE A3:** Generating site‐specific knowledge on non‐native tree species (NNT) in the risk assessment area in Step 5, list of parameters determining regeneration dynamics, competitiveness, and potential impacts of non‐native trees

Assessment of	Parameter	Reference
Establishment potential	Degradation	Sitzia et al. (2015)
	Disturbances	Sitzia et al. (2015)
	grazing intensity/ browsing intensity	Vor (2005)
	Intensity of human influence	Sitzia et al. (2015)
	light availability (canopy & understory cover, tree species composition)	Fanal et al. (2021), Meloni et al. (2016), Montgomery (2004)
	soil parameters (e.g., thickness of litter, pH, moisture)	Major et al. (2013)
Management options	estimate feasibility to implement management measures in a specific habitat	Booy et al. (2017), EC (2018)
	potential introduction pathways on site	McGeoch and Latombe (2016)
Potential impacts	abundance (number and cover) of seedlings and saplings	Dyderski and Jagodziński (2018), Major et al. (2013), Vor (2005)
	advance regeneration	Major et al. (2013), Vor (2005)
	age classes of trees	Bindewald et al. (2021), Vor (2005)
	dominance of NNT compared to other tree species	Branquart et al. ()
	heights of seedlings and saplings	Dyderski and Jagodziński (2018), Major et al. (2013), Vor (2005)
	natural regeneration in different tree stand types, including protected areas	Bindewald et al. (2021), Major et al. (2013), Verheyen et al. (2007)
	NNT density	Fanal et al. (2021)
	NNT vegetative propagation	Vor et al. (2015)
	tree species composition: abundance, species richness and evenness of all tree species	Dyderski and Jagodziński (2020), Dyderski and Jagodziński (2021), Vor et al. (2015)
Spread potential	NNT distance from propagule source	Jagodziński et al. (2018), Nygaard and Øyen (2017), Schmiedel et al. (2013)
	environmental (especially climate) data of the risk assessment area and the introduced range	Chakraborty et al. (2019)
	environmental (especially climate) data of the species native distribution	Chakraborty et al. (2019), Peterson et al. (2015)
	natural regeneration outside of planted sites	Carrillo‐Gavilan et al. (2012), Fernandes et al. (2016)
	tree species distribution, phytosociological background, assess the respective risk for the species due to climate change for suitability maps	Albrecht et al. (2019)
	Species presence/absence data	Ibáñez et al. (2009)
	Tree growth and reproduction data for forest growth and ecosystem models	Chakraborty et al. (2019)
